# Feasibility of the modified sequential organ function assessment score in a resource-constrained setting: a prospective observational study

**DOI:** 10.1186/s12871-017-0304-8

**Published:** 2017-01-26

**Authors:** Cornelius Sendagire, Michael S. Lipnick, Sam Kizito, Rebecca Kruisselbrink, Daniel Obua, Joseph Ejoku, Lameck Ssemogerere, Jane Nakibuuka, Arthur Kwizera

**Affiliations:** 1 Department of Anesthesia and critical care,, Makerere University College of Health Sciences, P.O. Box 7072, Kampala, Uganda; 2 San Francisco General Hospital, University of California San Francisco, 1001 Potrero, 3C24, San Francisco, CA 94110 USA; 3 McMaster University, 1280 Main St W, Hamilton, ON L8S 4L8 Canada; 4 Mulago National Referral Hospital, P.O. Box 7051, Kampala, Uganda

**Keywords:** Modified sequential organ function assessment, Mortality, Low and middle-income countries, Illness severity scoring system, Critical care

## Abstract

**Background:**

Sub-Saharan Africa has a great burden of critical illness with limited health care resources. We evaluated the feasibility and utility of the modified Sequential Organ Function Assessment (mSOFA) score in assessing morbidity and mortality in the National Referral Hospital’s intensive care unit (ICU) for one year.

**Methods:**

We conducted a prospective, observational cohort study on patients above 12 years of age admitted to the ICU at Mulago Hospital (Kampala, Uganda). All SOFA scores were determined at admission and at 48 h. We modified the SOFA score by replacing the PaO_2_/FiO_2_ ratio with SPO_2_/FiO_2_. The primary outcome was ICU mortality.

**Results:**

This ICU cohort of 118 patients had a mean age of 37 years and an ICU mortality rate of 46.6%. Non-survivors had higher initial (7.7 SD 3.8 vs. 5.5 SD 3.3; *p* = 0.007), mean (8.1 SD 3.9 vs 4.7 SD 2.6; *p* < 0.001) and highest mSOFA scores (9.4 SD 4.2 vs. 5.8 SD 3.2; *p* < 0.001), with an increase of 1.0 (SD 3.1) mSOFA on average after 48 h when compared to survivors (*p* < 0.001). The area under the receiver operating characteristic curves for each mSOFA category was: initial-0.68, mean-0.76, highest-0.76 and delta mSOFA-0.74. Multivariate logistic regression analysis showed no significant association between mSOFA scores and mortality.

**Conclusion:**

Our results confirm that calculation of the mSOFA score is feasible for an ICU population in a resource-limited country. More data are needed to test for an association between mSOFA and mortality.

## Background

Critical illness in low and middle income countries (LMICs) is a major component of the global burden of disease [1]. These countries have relatively younger patient populations than those of high income countries (HICs) [2, 3]. Despite this, ICU patients in LMICs still have more morbidity and mortality than those in HICs largely due to limited resources and the severity of co-morbid conditions [1–4].

In 1994, the European Society of Intensive Care Medicine (ESICM) committee developed the Sequential Organ Failure Assessment (SOFA) score 5, 6]. This score assesses the functionality of six organ systems (Table 1) [7]. It was validated as a tool for assessment of organ dysfunction and mortality prediction in ICU [8]. While simpler than many other tools used to predict ICU mortality, the application of SOFA still requires several lab tests (platelets, bilirubin, creatinine and blood gas analysis) [6, 8, 9]. The availability of such lab tests in LMICs is limited thereby limiting the widespread applicability of this score [1]. Some studies have validated modifications of the SOFA score in which certain lab tests were replaced with more readily available clinical measurements [10–12]. The SOFA score, for example, requires blood gas analysis for calculating the PaO_2_/FiO_2_ ratio [5]. However, a study in a HIC validated the use of SpO_2_/FiO_2_ ratio as a surrogate for the PaO_2_/FiO_2_ [10]. This modification was hypothesized to increase feasibility in settings where blood gas measurements are not regularly available. However, this modified SOFA score has not been validated in LMICs. In another study in a HIC, a modified SOFA score requiring only one laboratory measurement was validated to assess feasibility for critical care triage during pandemics [11]. In that study, Grissom et al. eliminated platelet count, substituted SpO2 for PaO2, replaced bilirubin with icterus and did bedside point-of-care creatinine testing. The only study to date from a low-income country was conducted in an urban hospital in Nepal and validated the use of the SOFA score but without any modification [12].

**Table 1:** Sequential Organ Function Assessment score

SOFA score	0	1	2	3	4
Respiratory					
PaO_2_/FiO_2_	>400	300–400	200–300	100–200	<100
SPO_2_/FIO_2_^a^	>301	221–301	142–220	67–141	<67
Coagulation					
Platelets 10^3^/mm^3^	>150	<150	<100	<50	<20
Liver					
Bilirubin (mg/dL)	<1.2	1.2–1.9	2.0–5.9	6.0–11.9	>12.0
Cardiovascular					
Hypotension	No	MAP	Dopamine	Dopamine	Dopamine
	hypotension	<70	</=5ug/kg/min or dobutamine (any dose)	>5ug/kg/min or norepinephrine </=0.1ug/kg/min	>15ug/kg/min or norepinephrine >0.1ug/kg/min
CNS					
Glasgow Coma	15	13–14	10–12	6–9	<6
Score					
Renal					
Creatinine (mg/dL)/urine output (ml/d)	<1.2	1.2–1.9	2.0–3.4	3.5–4.9 or <500	>5.0 or <200

*SPO*_2_: Peripheral saturation of oxygen; *FiO*_2_: Fraction of inspired Oxygen; *MAP*: Mean Arterial Pressure; *CNS*; Central Nervous System

^a^Modification to include SPO_2_/FIO_2_ as a replacement for PaO_2_/FiO_2_

Due to low specificity of the SIRS criteria, the most recent sepsis guidelines have incorporated SOFA score into the new sepsis definition [13, 14]. Unlike SIRS criteria however, the SOFA requires more laboratory evaluations which limit its feasibility in LMICs [9].

With a paucity of literature aimed at addressing critical care in LMICs, we conducted a study to determine the feasibility and utility of using a modified SOFA (mSOFA) Score for assessing organ dysfunction and predicting mortality in a tertiary hospital ICU of a low-income country.

## Methods

### Study area and setting

We conducted a prospective, observational cohort study for one year (February, 2014 – January, 2015) in the ICU of Mulago Hospital (Kampala, Uganda). Mulago Hospital is a 1500-bed teaching hospital and the national referral hospital for Uganda. The hospital’s inpatient census often exceeds 2000 and has a large proportion of critically-ill patients [15]/ The hospital’s ICU has eight beds although only four beds are equipped with ventilators. The ICU is staffed by two intensivists, one senior resident, one to two anesthesia residents per day, and nurses. It has a nurse to patient ratio of 1:2 – 1:4. Complete blood counts, as well as liver and renal function tests are routinely available; however, blood gas analysis is rarely available. The ICU admits between 200–300 critically-ill patients annually with an ICU mortality of approximately 40% [16].

### Study population

Study approval was granted by the Makerere University School of Medicine Research and Ethics Committee. A written informed consent/assent was obtained from next of kin prior to enrollment or a waiver of consent was obtained for those without next of kin. All patients >12 years of age admitted to the Mulago Hospital ICU for more than 24 h were enrolled. We chose a lower limit of 13 years because the SOFA score has not been validated among patients <13 years [5]. The exclusion criteria were admission for low risk monitoring, death or discharge in the first 24 h and no outcome at the end of the study.

### Study procedure

We collected demographic data, all vital signs, complete blood count, as well as liver and renal function tests for each patient at admission and at 48 h after admission. Blood gas analysis was to be done with the ABL 80 Flex model in the ICU. The mSOFA score, ranging from 0 (normal organ function) to 24 (worst organ dysfunction), was calculated on admission (T_0_) and at 48 h (T_48_). Patients were then followed until ICU discharge or the end of the study period. In calculating the mSOFA score, the worst values for each parameter in the 24-h period were used. The SPO_2_/FiO_2_ ratio was used instead of PaO_2_/FiO_2_ ratio, and the remaining parameters were used based on the calibration as shown in Table 1. We selected the range cutoffs shown in Table 1 as these ranges had been validated in two prior studies. For patients on nasal cannula oxygen, we estimated FiO_2_ by multiplying the liter flow per minute by 0.03 and adding that to 0.21. For a single missing data point, a replacement was estimated by calculating the mean of the sum of the results immediately preceding and following the missing value. The total mSOFA score was calculated as the sum of the mSOFA score at admission and at 48-h. The initial mSOFA score was the average T_0_ of all patients. The mean mSOFA score was defined as the average of mSOFA score over the two days of calculation of the mSOFA while in the ICU. The highest recorded SOFA score was also recorded as the highest between the two recording times. The delta SOFA score was defined as the difference between T_0_ and T_48_. After discharge or death, data were crosschecked for completeness and accuracy with patient’s chart prior to data entry by a trained staff member.

### Statistical analysis

The primary outcome was ICU mortality or discharge. We compared continuous variables of the survivors and non-survivors using the student *t*-test with a significant *p*-value of <0.05. We used the chi-square test for categorical variables with a significant *p*-value of <0.05. Odd ratios with 95% confidence interval were computed using a multivariate logistic regression model with ICU mortality as the dependent variable. The predictive ability of the SOFA scores was evaluated using receiver operating characteristic (ROC) curve analysis generated on STATA 12 (Statacorp College Station, TX). The area under the receiver operating characteristic curve (AUROC) was used to compare the discriminatory power of the scoring system, with an AUC 1.0 considered perfect discrimination and 0.5 considered equal to chance. Numerical continuous data were summarized as means, standard deviations for the normally distributed data, and as medians and interquartile ranges for the non-normally distributed data. Categorical data were summarized with percentages and proportions.

## Results

**Fig. 1:**
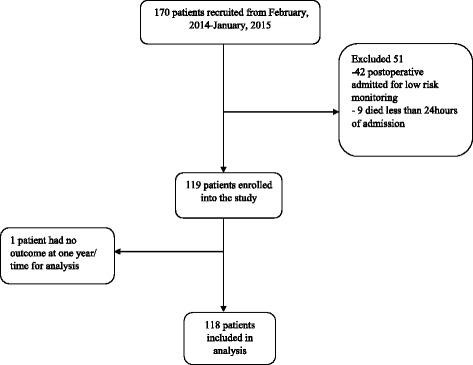
Enrolment

From February 20, 2014 to January 31, 2015 we screened 201 patients, and enrolled 170. Subsequently, 51 patients were excluded. Of these, 42 of them were admitted for only 24-h of postoperative monitoring, nine died within 24 h of admission and only one had no outcome at the end of the study (Fig. 1). There were nine non-adults (ages 13–18) in the cohort, all of whom had informed consent obtained from their appropriate family members. We thus analyzed 118 patients (58% male) with a mean (SD) age of 37 (17) years and an ICU mortality rate of 46.6%. During the study period, PaO_2_ values were missing in 90% of the study patients while there was no missing value for bilirubin, creatinine and platelet count. The median length of stay in the ICU was six days (IQR; 3–10) while the median survival time within the ICU was 12 days (95% CI 6.38–17.62). Sepsis prevalence according to the SIRS criteria was 50% on admission to the ICU. Table 2 summarizes the demographics and initial clinical characteristics of the study cohort.

**Table 2:** Demographics and clinical characteristics of 118 critically patients admitted to mulago hospital icu from February 2014 to January 2015

Characteristics	All	Survivors	Non-Survivors	*P* ^a^
Age (years); mean (SD)	37.7 (17.5)	37.7 (17.4)	37.7 (17.7)	0.99
Sex				
Male (%)	68 (57.6)	41 (66.7)	25 (47.3)	0.033
Source (%)				
Emergency room	33 (28)	19 (30.2)	14 (25.4)	
Operating room	21 (17.8)	15 (23.8)	6 (10.9)	
PACU	2 (1.7)	2 (3.2)	0 (0.0)	
Obstetric theatre	3 (2.5)	0 (0.00)	3 (5.5)	
Medical ward	25 (21.2)	9 (15.9)	15 (27.3)	
Surgical ward	11 (9.3)	6 (9.5)	5 (9.1)	
Obstetric-gynecology ward	7 (5.9)	2 (3.2)	5 (9.1)	
Other hospital	16 (13.6)	9 (14.3)	8 (12.7)	0.117
Medical Admissions (%)	40 (33.9)	20 (31.8)	20 (36.4)	0.597
HIV status (%)				
Positive	12 (10.1)	6 (9.5)	6 (10.8)	
Negative	88 (74.6)	48 (77.8)	41 (73.6)	
Unknown	18 (15.3)	8 (12.7)	9 (16.2)	0.664
Mechanical Ventilation (%)	100 (84.8)	47 (47.0)	53 (53.0)	0.001
Ventilation time (SD) (hours)	159.07 (247.87)	156.05 (227.95)	162.53 (270.99)	0.888
Vasopressor use (%)	28 (23.7)	4 (14.2)	24 (85.7)	0.008
Length of stay (Days);mean (SD)	10.2 (12.8)	12.5 (13.8)	7.5 (11.1)	0.033
No. of patients (%)	118	63 (53.4)	55 (46.6)	

*PACU*: Post anaesthetic care unit; *SD*: Standard deviation; *HIV*: Human immunodeficiency virus; *ICU*: Intensive care unit; *ABG*: Arterial blood gas

*P* ^a^ values calculated using unpaired *t* tests, Mann–Whitney *U* tests, or chi-square tests where appropriate

The initial, mean, highest and delta mSOFA scores (SD) were: 6.5 (3.7), 6.3 (3.7), 7.5 (4.1) and 0.4 (3.2) respectively. Non-survivors had significantly higher initial, mean and higher mSOFA scores than survivors: 7.7 vs. 5.5 (*p* = 0.001), 8.1 vs. 4.7 (*p* < 0.001) and 9.4 vs. 5.8 (*p* < 0.001) respectively. We also found that while the initial mSOFA score decreased by 1.7 in survivors; that of non-survivors increased by 1.0 after 48 h (*p* < 0.001). On admission, only the respiratory and cardiovascular mSOFA scores of non-survivors were significantly higher than those of survivors (Table 3). The same trend was observed at 48 h, however, the central nervous system and renal mSOFA scores were also significantly higher in non-survivors.

**Table 3:** Calculated sofa scores and comparisons of 118 critically ill-patients admitted to mulago hospital icu from February 2014 to January 2015

SOFA score	All	Survivors	Non-survivors	*P* ^a^
SOFA-0 mean (SD)				
SOFA-RS	1.6 (1.2)	1.3 (1.2)	1.9 (1.2)	0.007
SOFA-CVS	0.6 (1.2)	0.2 (0.7)	1 (1.6)	0.001
SOFA-CNS	2.8 (1.1)	2.8 (1.2)	2.8 (1.1)	0.66
SOFA-Liver	0.3 (0.8)	0.2 (0.7)	0.4 (0.8)	0.168
SOFA-coagulation	0.3 (0.7)	0.3 (0.6)	0.4 (0.7)	0.364
SOFA-renal	1 (1.5)	0.8 (1.4)	1.2 (1.6)	0.115
Total SOFA	6.5 (3.7)	5.5 (3.3)	7.7 (3.8)	0.001
SOFA-48 mean (SD)				
SOFA-RS	1.2 (1.2)	0.7 (0.9)	1.8 (1.4)	<0.001
SOFA-CVS	0.7 (1.4)	0.1 (0.4)	1.4 (1.8)	<0.001
SOFA-CNS	2.6 (1.4)	2 (1.3)	3.4 (1.0)	<0.001
SOFA-Liver	0.2 (0.6)	0.2 (0.6)	0.3 (0.6)	0.379
SOFA-coagulation	0.3 (0.7)	0.3 (0.6)	0.4 (0.7)	0.620
SOFA-renal	1 (1.5)	0.7 (1.4)	1.4 (1.6)	0.007
Total SOFA	6.0 (4.3)	3.9 (2.6)	8.5 (4.6)	<0.001
Mean SOFA (SD)	6.3 (3.7)	4.7 (2.6)	8.1 (3.9)	<0.001
Highest SOFA (SD)	7.5 (4.1)	5.8 (3.2)	9.4 (4.2)	<0.001
Delta SOFA (SD)	0.4 (3.2)	-1.7 (2.7)	1.0 (3.1)	<0.001

*SOFA*: Sequential organ function assessment; *RS*: Respiratory system; *CVS*: Cardiovascular system; *CNS*: Central nervous system

*P* ^a^ values calculated using unpaired *t* tests, Mann–Whitney *U* tests

**Fig. 2:**
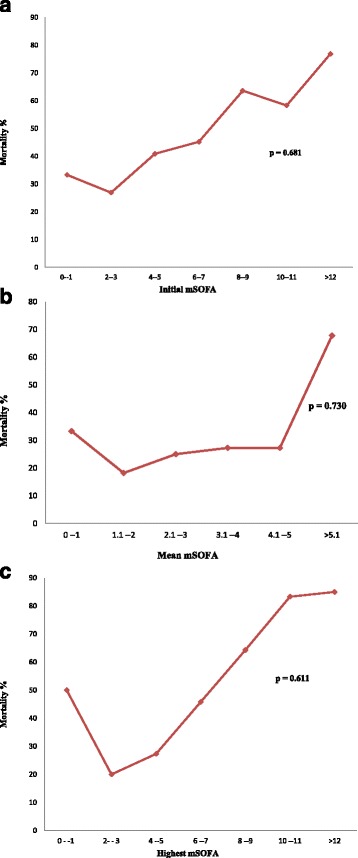
Association of Initial, Mean and Highest mSOFA with Mortality. These graphs demonstrate a direct proportionality relationship between mSOFA scores with mortality even though there was no significant association in the multivariate analysis. **a** shows the Initial mSOFA score association with mortality. **b** shows the Mean mSOFA score association with mortality. **c** shows the highest mSOFA score association with mortality

The adjusted odds ratios of the mSOFA scores for mortality were not statistically significant despite trends showing direct proportionality with mortality as seen in Fig. 2 and neither was there a statistical difference in their slopes found. The 48-h change in mSOFA score also had no significant association with mortality *p*-value = 0.61.

**Fig. 3:**
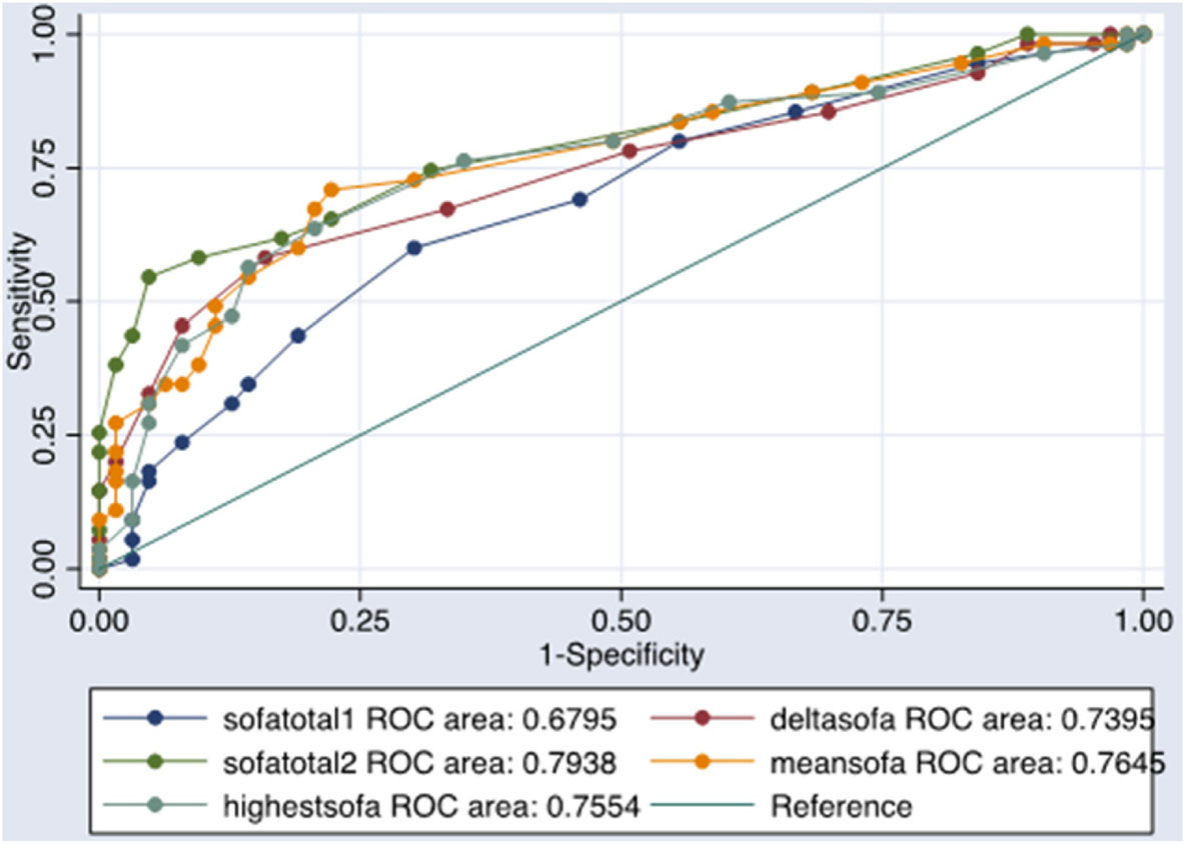
Receiver Operating Curves for mSOFA prediction of mortality. The mSOFA demonstrated a poor to fair prediction of mortality. The initial mSOFA score is represented by Sofatotal1. It showed the lowest predictive power of all categories of the mSOFA. The mSOFA at 48 h, represented by Sofatotal2, had the highest predictive power followed by the mean, highest and delta mSOFA scores

Using AUROC for discriminating mortality, the initial mSOFA score had the lowest predictive power with an AUC of 0.68 (95% CI, 0.58-0.77) while the mSOFA at 48 h had the highest predictive power of 0.79 (95% CI, 0.71–0.87). The mean, highest and delta mSOFA scores demonstrated a fairly accurate prediction with AUROC: 0.76 (95% CI, 0.68–0.85), 0.76 (95% CI, 0.66–0.85), and 0.74 (95% CI, 0.65–0.84) respectively (Fig. 3 and Table 4). We also found that the mean mSOFA score AUC was significantly higher than that of the initial mSOFA with a *p* value of 0.0003.

**Table 4:** Mortality predictive power of the msofa score for 118 critically ill-patients admitted to mulago hospital icu from February 2014 to January 2015

mSOFA	AUROC	95% Confidence interval
Initial	0.68	0.58–0.78
48 h	0.79	0.71–.88
Mean	0.76	0.68–0.85
Highest	0.76	0.67–0.85
Delta	0.74	0.65–0.84

## Discussion

The present study is the first in Sub-Saharan Africa to determine the feasibility and utility of modifying the SOFA score for evaluating organ dysfunction and critical care mortality in a resource-limited ICU. In our study we were able to calculate mSOFA for all patients even though we were unable to prove that it is a good predictor of mortality. Since 90% were missing PaO_2_ values; majority of patients in our study did not have data required to calculate a traditional SOFA score.

The use of simple, inexpensive and rapid methods to assess illness severity and predict outcomes is important in all clinical settings, especially those in low-income countries where resources are limited and illness severity is high [1, 4, 6, 11]. Recently the specificity of SIRS criteria for sepsis has come into question and led to the development of new sepsis definitions that now include the SOFA score [13, 14, 17, 18]. Although the Third Consensus Definitions for Sepsis and Septic Shock includes a discussion of quick SOFA (qSOFA) scores, the vast majority of efforts to simplify or validate scoring tools (like SOFA) take place almost exclusively in high-income countries.

The initial mSOFA score for our study was less than that of a study from HIC and the only other study from a LIC; Nepal (6.5 vs. 7.1 vs. 7.9 respectively) [7, 12]. The Nepalese study is the only prior study of SOFA scores in a LIC and utilized the original SOFA score in an urban hospital (six bed ICU) [12]. That study found significant predictive value of SOFA scores for mortality using univariate analysis. Similar to our study, Acharya et al. also had a relatively young patient cohort (mean age 34 years) with a high mortality rate (40%). Significant differences between our studies exist and include the relatively smaller sample size, better hospital resources (e.g. regular availability of blood gas analyses) and inclusion only of patients who met the SIRS criteria for the Nepalese study. Our initial mSOFA was lower than that from Acharya despite our population having higher mortality rate overall. This suggests that our SpO2 modification may underestimate the respiratory variable of the mSOFA score as discussed in greater detail below.

The results of prior studies of modified SOFA scores in HICs may not be applicable in LICs due to marked differences in clinical environments and patient populations [4, 11]. In the current study we used the SPO_2_/FiO_2_ ratio as a surrogate for the PaO_2_/FiO_2_ in calculating the SOFA score, a modification previously validated but only in HICs [10, 11]. Overall, the PaO_2_ values were missing in a very large percentage of our patient despite the fact that 48% had at least one blood gas analysis done during the ICU stay. This 48% was still a relatively larger proportion of patients with blood gas done than hypothesized, but due in large part to a concurrent observational study that provided cartridges for the blood gas machine stationed in the ICU. We found that the PaO_2_ values were missing even in those who had blood gas analysis done because it was done outside the recording times: T_0_ and T_48_.

Compared to ICUs of HICs, those in LICs have a mortality rate nearly four times higher largely due to inadequate resources [1, 2, 4, 12, 16, 19]. The mortality rate in our study was relatively higher than that found in a prior study done in the same ICU by Kwizera et al. and Acharya’s study in Nepal: 47 vs. 43 vs. 40% respectively [12, 16]. Kwizera et al’s study had no exclusion criteria unlike our study, which could have led to their slightly lower mortality rate. The Nepalese study was conducted in an ICU that was better resourced than our own and was conducted on patients who met the SIRS criteria. Since positive SIRS criteria has been associated with a higher mortality rate [13], it is highly likely that this was the reason their mortality was nearly as high as that found in our study.

We found that all patients in the present study had mSOFA scores more than twice the designated cut off for organ dysfunction as outlined by the The Third International Consensus Definitions for Sepsis and Septic Shock [14]. Similar to other studies, the mSOFA scores of non-survivors in our cohort were significantly higher than those in survivors [5, 7, 8, 12, 19, 20]. We also found that the initial mSOFA score had the lowest AUROC compared to the mean, highest and delta mSOFA scores, similar to prior investigations [8, 12, 21]. With exception of the initial mSOFA, the other mSOFAs demonstrated fairly accurate prediction however the confidence intervals were wide and thus ought to be interpreted with caution. This was also supported by the finding that the mean mSOFA had a significantly higher predictability than the initial mSOFA. Our univariate analysis did show predictive value for the initial mSOFA yet this relationship did not hold true with multivariate analysis. Of note, the prior study in Nepal undertook only univariate analysis that showed significant associations [12]. Even though we found no significant association between the mSOFA and mortality, Fig. 2 shows a linear relationship if a line of best fit was drawn for each of the mSOFA categories. We still however, did not find any statistical significance of any of the slopes in any combination. We believe this was due to the small sample size such that occurrence of mortality in any of the ranges we used for each mSOFA category resulted into a bigger percentage of that category than would have been otherwise.

Prior studies have found that SOFA score is most predictive in the first 48 h and can be used to assess degree of organ dysfunction, mortality and response to therapy throughout admission [8]. Although our results were unable to corroborate all of these findings, this study provides evidence that further validation and modification of the most recent sepsis definitions is feasible in LICs and warranted. The utility of a simple validated tool for assessing and predicting outcomes in LICs would have far-reaching applicability that extends beyond patient care and also includes future research to improve outcomes in these settings.

There were several limitations with the present study. The first and most significant was low power due to small sample size. Due to limited access to medical records and limited study personnel, we were unable to enroll and analyze data for patients admitted for less than 24 h. This resulted in the exclusion of a large number of potential study subjects. Another significant limitation of the current study may be attributable to the modification of SOFA scores using SpO_2_ in our setting. There are several reasons the SpO_2_/FiO_2_ calculation may not accurately reflect severity of pulmonary dysfunction. For example, in order to be categorized in the sickest mSOFA cohort by SpO_2_, a patient would need to have a SpO2 of <67% while on 100% FiO_2_. Only three patients in our cohort met this criterion. Our use of SpO_2_ likely underestimated severity of pulmonary dysfunction. For example, a patient with an SpO_2_ of 90% on 100% FiO2 would be given 3 points using the mSOFA (90/1 = 90; Table 3). Using the original SOFA score and assuming that same patient has a PaO_2_ of ~60, the patient would be given 4 points (60/1 = 60; Table 3). An additional significant limitation of SpO_2_/FiO_2_ calculations in our setting is the lack of reliable oxygen sources. For example, tank and wall oxygen frequently are less than 100% FiO2 (anecdotally as low as 70%). This could result in significant overestimation of severity of pulmonary dysfunction. In future investigations, the investigation of multiple SpO_2_ respiratory thresholds may yield improved predictive value for the mSOFA Score. An additional limitation that we must recognize due to our single site design is the possibility for site bias. We performed the mSOFA score on only two occasions, 48 h apart, limiting the information on organ dysfunction assessment over the length of stay with treatment. The generalizability to austere environments outside our study setting is challenging, as further investigation and more robust study resources are needed.

## Conclusion

Our results confirm that calculation of the mSOFA score is feasible for an ICU population in a low-income country. Although the present study did not demonstrate a definitive relation between mSOFA and mortality, larger studies are needed to assess the discriminative power of mSOFA for predicting organ dysfunction and mortality in resource-limited settings. Such data may be able to help better characterize critical illness disease burden and the triage of limited resources.

## List of abbreviations

APACHE: Acute Physiologic And Chronic Health Evaluation
CNS: Central Nervous System
ESICM: European Society of Intensive Care Medicine
FiO_2_: Fraction of inspired Oxygen
GSK: GlaxoSmithKline
HICs: High Income Countries
ICU: Intensive Care Unit
IQR: Interquartile Range
LMICs: Lower Middle Income Countries
mSOFA: modified Sequential Organ Function Assessment Score
PaO_2_: Arterial Partial pressure of Oxygen
qSOFA: quick Sequential Organ Function Assessment
SAPS: Simple Acute Physiologic Score
SD: Standard Deviation
SIRS: Systemic Inflammatory Response Syndrome
SOFA: Sequential Organ Function Assessment
SPO_2_: Peripheral Saturation of Oxygen
T_0_: mSOFA at admission (initial SOFA Score)
T_48_: mSOFA at 48 h
